# Adsorption of nitrophenol onto a novel Fe_3_O_4_-κ-carrageenan/MIL-125(Ti) composite: process optimization, isotherms, kinetics, and mechanism

**DOI:** 10.1007/s11356-023-25678-2

**Published:** 2023-02-11

**Authors:** Eman M. Abd El-Monaem, Abdelazeem S. Eltaweil, Gehan M. El-Subruiti, Mohamed S. Mohy-Eldin, Ahmed M. Omer

**Affiliations:** 1 Chemistry Department, Faculty of Science, Alexandria University, Alexandria, Egypt; 2 Polymer Materials Research Department, Advanced Technology and New Materials Research Institute (ATNMRI), City of Scientific Research and Technological Applications (SRTA-City), P. O. Box: 21934, New Borg El-Arab City, Alexandria, Egypt

**Keywords:** κ-Carrageenan, MOF (MIL-125(Ti)), Magnetic composite, Nitrophenol, Adsorption, Reusability

## Abstract

Water pollution is a dreadful affair that has incessantly aggravated, exposing our planet to danger. In particular, the persistent nitro aromatic compound like nitrophenols causes anxiety to the researchers due to their hazardous impacts, excessive usage, and removal difficulty. For this purpose, a novel multi-featured composite was constructed based on κ-Carrageenan (κ-Carr), MOF (MIL-125(Ti)), and magnetic Fe_3_O_4_ for efficient adsorptive removal of o-nitrophenol (o-NP). Interestingly, BET measurements revealed the high surface area of Fe_3_O_4_-κ-Carr/MIL-125(Ti) of about 163.27 m^2^/g, while VSM showed its excellent magnetic property (20.34 emu/g). The comparison study pointed out the synergistic effect between Fe_3_O_4_, κ-Carr, and MIL-125(Ti), forming a composite with an excellent adsorption performance toward o-NP. The adsorption data obeyed pseudo-second-order kinetic model, and Freundlich isotherm model was better fitted than Langmuir and Temkin. Furthermore, Langmuir verified the supreme adsorption capacity of o-NP onto Fe_3_O_4_-κ-Carr/MIL-125(Ti) since the computed q_max_ reached 320.26 mg/g at pH 6 and 25 °C. Furthermore, the XPS results postulated that the adsorption mechanism pf o-NP proceeded via H-bonding, *π*-*π* interaction, and electron donor–acceptor interactions. Interestingly, Fe_3_O_4_-κ-Carr/MIL-125(Ti) composite retained good adsorption characteristics after reusing for five cycles, suggesting its viable applicability as an efficient, renewable, and easy-separable adsorbent for removing nitro aromatic pollutants.

**Supplementary Information:**

The online version contains supplementary material available at 10.1007/s11356-023-25678-2.

## Introduction

Water pollution by the fatal aromatic compounds is swiftly exacerbated, threatening humanity’s existence (Das et al. 2021; Kassem et al. 2021; Priyadarshi et al. 2022). Nitrophenol is one of the most pernicious aromatic compounds since it is widely applied in diversified industries such as dyes, insecticides, medicines, and petrochemicals (Abdelfatah et al. 2021a; Liu et al. 2020). Consequently, colossal amounts of nitrophenol have been disposed into waterbodies, causing deleterious impacts on human health, such as damage to kidneys and liver, blurred vision, systemic poisoning, and mouth irritation (Benmaati et al. 2022; Ma et al. 2019). In addition to the dangerous effects on the environment, the presence of the nitro group in nitrophenol enhances its stability in soil and water bodies (Ewis et al. 2022). Hence, several remediation techniques have been fostered to get rid of these detrimental contaminants from water bodies, such as membrane separation, coagulation, electrolysis, ion exchange, and adsorption (Abdelfatah et al. 2021a, Abdelfatah et al. 2021b, Eltaweil et al. 2022a, Karim et al. 2019, Li et al. 2021, Sadoon and M-Ridha 2019, Saha et al. 2022, Tran et al. 2021, Wu et al. 2021). The former technique has been applied more widely than the other techniques owing to its simplicity, low cost, high-efficacy, and low-energy consumption (Abdelfatah et al. 2022; Deb et al. 2021; Gomaa et al. 2022b; Mokhtar et al. 2020; Raval et al. 2021; Zhao et al. 2021).

Carrageenan is an anionic polysaccharide polymer extracted from red seaweed (Yu et al. 2019). Carrageenan involves sulfate groups, and it is classified into theta, beta, iota, lambda, alpha, and kappa based on the position and numbers of the attached sulfate groups onto the carrageenan skeleton (Sharma et al. 2022). Interestingly, κ-Carr is vastly utilized in the food industries since it has remarkable thickening, stabilizing, and gelling properties (Ammar et al. 2021). Furthermore, the eco-friendly, availability, biodegradability, and biocompatibility characteristics of κ-Carr render it a promising adsorbent for removing contaminants (Li et al. 2019). In spite of the remarkable advantages of κ-Carr, it still suffers some limitations, including an inferior gel strength and poor environmental stability (Lapwanit et al. 2018). Thence, several attempts have been implemented to overcome these flaws, including modification of κ-Carr with carbon materials, magnetic nanoparticles, zeolite, and other polymers (Duman et al. 2019, 2016; Huang et al. 2022; Mittal et al. 2020). Notably, it was reported an enhancement in the κ-Carr properties by impregnating metal–organic frameworks (MOFs) into its matrix (Klongklaew and Bunkoed 2021). Nevertheless, there is a scarcity of research papers that involves the fabrication of κ-Carr/MOF composites.

Strikingly, the applications of MOFs have been raised day-by-day in diversified fields such as drug delivery, solar cell, gas storage, batteries, and especially in water remediation. (Lazaro and Forgan 2019, Shen et al. 2021; Xu et al. 2022). Due to their excellent chemical and thermal stability, high surface area, porous structure, water stability, and ease of functionalization, MOFs have gained vast concern as propitious adsorbents (Abd El-Monaem et al. 2022). Titanium-based MIL-125 is a shining member of the remarkable MIL family that possesses excellent adsorption performance owing to excellent chemical stability, redox potential, and thermal stability (Fatima et al. 2020). Interestingly, MIL-125(Ti) has revealed an enhanced adsorption performance toward pharmaceutical residues, organic dyes, and heavy metals (Jiang et al. 2021; Liang et al. 2018; Liu et al. 2021; Omer et al. 2021). However, the difficult separation of MIL-125(Ti) after the adsorption process with the traditional techniques is a big obstacle. To overcome this disadvantage, incorporating magnetic nanoparticles in the adsorbent matrix enable easy separation of them after adsorption. One of the most extensively utilized magnetic nanoparticles is magnetite due to its ease of synthesis, biocompatibility, high surface area, and abundant active sites (Attia et al. 2022; Toto et al. 2022). Despite the construction of various composites from MIL-125(Ti) with many polysaccharides, there are no study reported the fabrication of MIL-125(Ti)/κ-Carr composite and the evaluation of its adsorbability toward organic pollutants or any other water contaminants.

Herein, we adopted a developed avenue to foster the adsorption performance of κ-Carr through its combination with MIL-125(Ti). Furthermore, to overcome the separation difficulty, κ-Carr/MIL-125(Ti) composite was decorated by magnetic nanoparticles (Fe_3_O_4_). Assorted characterization tools were utilized to infer the successful fabrication of Fe_3_O_4_-κ-Carr/MIL-125(Ti) and study its chemical and physical properties. The adsorbability of Fe_3_O_4_-κ-Carr/MIL-125(Ti) composite was examined in the adsorption of o-nitrophenol (o-NP) from an aqueous solution. Besides, the reusability of Fe_3_O_4_-κ-Carr/MIL-125(Ti) composite was confirmed by executing the recyclability test for five cycles. More importantly, the mechanism of the o-NP adsorption onto Fe_3_O_4_-κ-Carr/MIL-125(Ti) composite was understood thoroughly based on XPS results.

## Experimental part

### Materials

Ferrous chloride tetrahydrate (FeCl_2_.4H_2_O), glutaraldehyde, and ethanol were supplied from Aladdin Industrial Corporation, China. Ammonium solution (NH_4_OH), 1,4-benzene dicarboxylic acid (BDC), and N,N dimethyl formamide (DMF) were purchased from Sinopharm Chemical Reagent, China. κ-Carrageenan, ferric chloride hexahydrate (FeCl_3_.6H_2_O), o-nitrophenol, and titanium tetraisoproproxide (Ti(O-iPr)_4_) were provided from Sigma-Aldrich, USA.

### Fabrication of Fe_3_O_4_ nanoparticles

Fe_3_O_4_ was prepared as reported in the author’s previous study (Eltaweil et al. 2021). Under the N_2_ atmosphere, the specific amounts of FeCl_3_.6H_2_O and FeCl_2_.4H_2_O were dissolved into 500 mL double-distilled water. Then, ammonium solution was dropped slowly into the Fe^2+^/Fe^3+^ solution until pH reached 10. The resultant solution was stirred at 80 °C for 9 min. Finally, the obtained Fe_3_O_4_ particles were collected via a magnet, washed, and dried at 70 °C.

### Fabrication of MIL-125(Ti)

MIL-125(Ti) was prepared as follows: 995 mg BDC was dissolved into 25 mL DMF under magnetic stirring for 15 min. Then, 1.35 mL Ti(O-iPr)_4_ was dipped into the BDC solution and kept under vigorous stirring for 1 h. Next, Ti/BDC solution was poured into a 100 mL autoclave and heated at 130 °C for 24 h. Ultimately, the obtained solid was collected by centrifugation, washed, and dried at 80 °C for 24 h (Yang et al. 2018).

### Fabrication of Fe_3_O_4_-κ-Carr/MIL-125(Ti) composite

**Scheme 1:**
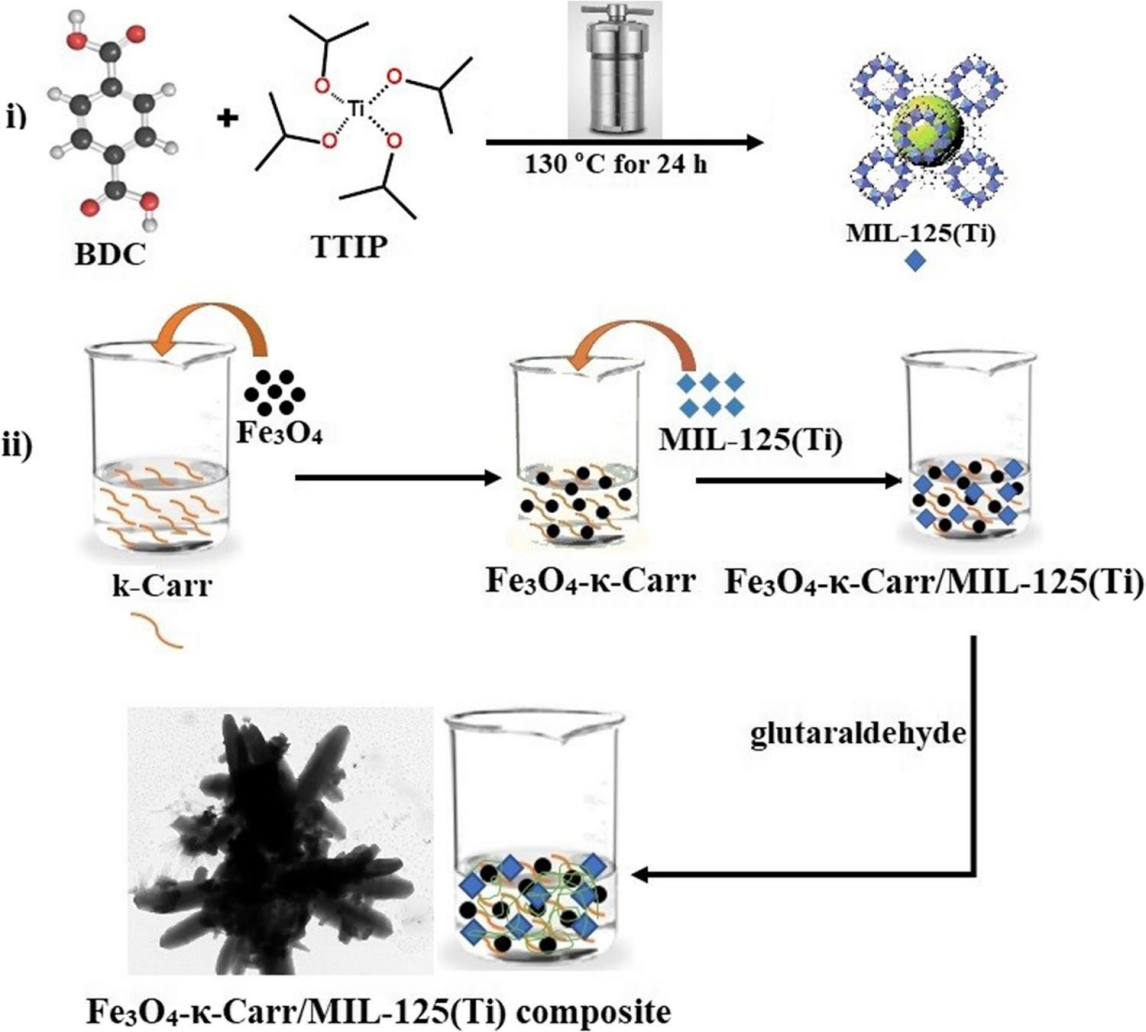
The preparation method of Fe_3_O_4_-κ-Carr/MIL-125(Ti)

Fe_3_O_4_-κ-Carr/MIL-125(Ti) composite was prepared as follows: a particular amount of κ-Carr was dissolved into 25 mL dist. H_2_O at 70 °C. Then, 0.02 g Fe_3_O_4_ was dipped into κ-Carr solution and kept under stirring for 1 h, allowing the magnetic nanoparticles to diffuse into κ-Carr layers. MIL-125(Ti) was added bit-by-bit to Fe_3_O_4_/κ-Carr composite under vigorous stirring, followed by adding 6 mL of glutaraldehyde. After 1 h, Fe_3_O_4_-κ-Carr/MIL-125(Ti) composite was collected, washed, and dried at 50 °C for 24 h. Fe_3_O_4_-κ-Carr/MIL-125(Ti) composite was prepared with three mass ratios of κ-Carr/MIL-125(Ti) = 1:1, 1:3, and 3:1 (Scheme 1).

### Characterization

Fe_3_O_4_-κ-Carr/MIL-125(Ti) composite and its components were characterized by Fourier transform infrared spectra (FTIR; NEXUS-670), Brunauer–Emmett–Teller method (BET; Tristar II 3020), X-ray diffractometer (XRD; MAC Science M03XHF), zeta potential (ZP; Malvern-UK), X-ray photoelectron spectroscopy (XPS; Escalab 250Xi), vibrating-sample magnetometer (VSM; Lakeshore), scanning electron microscope (SEM; Hitachi-S4800), and transmission electron microscope (TEM; JOEL, 110 kV).

### Batch adsorption

The optimum conditions to adsorb o-NP onto Fe_3_O_4_-κ-Carr/MIL-125(Ti) composite were defined in a batch mode. A series of the o-NP adsorption process proceeded at a pH range from 2 to 10 to identify the optimum pH. Furthermore, the impact of the Fe_3_O_4_-κ-Carr/MIL-125(Ti) dose on the adsorption aptitude of o-NP was determined at a dose range from 0.005 to 0.02 g. Moreover, the influence of the temperature on the removal efficiency of o-NP was examined at a temperature range from 25 to 55 °C. Finally, the impact of the initial concentration of o-NP was studied at a concentration range from 50 to 200 mg/L. The residual concentration of o-NP was measured according to the standard methods to examine water and wastewater (Rice et al. 2012) using UV–Vis spectrophotometer (PG 82 + , UK) at 344 nm, then the adsorption capacity and the removal % of o-NP were calculated by the following equations:

1 $$\mathrm{R \%}=\frac{{\mathrm{C}}_{0 }-{\mathrm{C}}_{\mathrm{t}}}{{\mathrm{C}}_{0}} \times 100$$

2 $${\mathrm{q}}_{\mathrm{e}}=\frac{{(\mathrm{C}}_{0}-{\mathrm{C}}_{\mathrm{t}})\times \mathrm{V}}{\mathrm{m}}$$

where *C*_0_ and *C*_*t*_ symbolize the initial concentration of o-NP and the concentration at time *t*, respectively. *m* and *V* symbolize the mass of Fe_3_O_4_-κ-Carr/MIL-125(Ti) and the volume of o-NP solution, respectively.

### Recyclability test

To evaluate the regeneration potential of Fe_3_O_4_-κ-Carr/MIL-125(Ti) composite, the used composite was separated after the o-NP adsorption and subsequently soaked into 25 mL 1 M NaOH under magnetic stirring to desorb o-NP from its surface. Then, the recycled composite was washed with distilled H_2_O and utilized in the next cycle, repeating this adsorption/desorption cycle five times.

### Ionic strength test

The influence of the ionic strength on the o-NP adsorption aptitude was assessed as follows: a specific weight of NaCl (0.2–1.0 mol/L) was soaked in 20 mL of o-NP at pH 6. Then, 10 mg of Fe_3_O_4_-κ-Carr/MIL-125(Ti) was added to o-NP/NaCl solution under stirring. After 60 min, a sample was withdrawn and measured to determine the concentration of the un-adsorbed o-NP.

## Results and discussion

### Characterization of Fe_3_O_4_-κ-Carr/MIL-125(Ti) composite

#### Morphology study

**Fig. 1:**
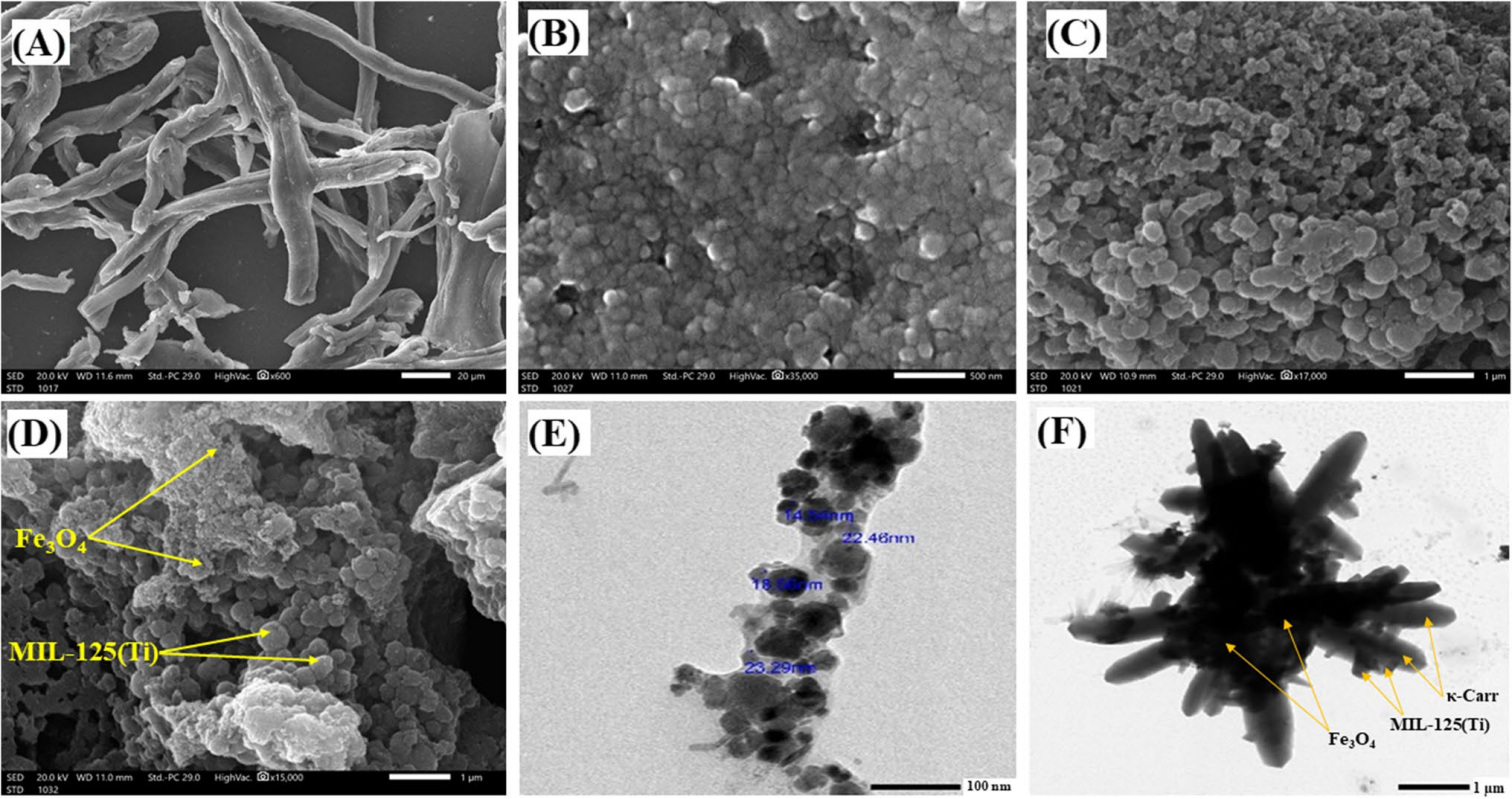
SEM images of **A** κ-Carr, **B** Fe_3_O_4_, **C** MIL-125(Ti), and **D** Fe_3_O_4_-κ-Carr/MIL-125(Ti) and TEM images of **E** Fe_3_O_4_ and **F** Fe_3_O_4_-κ-Carr/MIL-125(Ti)

SEM was utilized to identify the morphology of the as-synthesized Fe_3_O_4_-κ-Carr/MIL-125(Ti) composite and its pure components. The SEM image of κ-Carr (Fig. 1A) reveals its fiber-like morphology with a smooth surface, while the SEM image (Fig. 1B) showed the aggregated spherical particles of Fe_3_O_4_ in nanosize. Such aggregation is most likely due to the magnetic nature of Fe_3_O_4_. Furthermore, the SEM image of MIL-125(Ti) depicts a quasi-spherical morphology with irregular size (Fig. 1C). SEM of Fe_3_O_4_-κ-Carr/MIL-125(Ti) composite suggested the incorporation of Fe_3_O_4_ and MIL-125(Ti) particles into κ-Carr (Fig. 1D).

The TEM image (Fig. 1E) exhibited the quasi-spherical morphology of Fe_3_O_4_ with an average particle size of about 19.71 nm. In addition, the image revealed an aggregation of Fe_3_O_4_ particles owing to their magnetic nature. The TEM image of Fe_3_O_4_-κ-Carr/MIL-125(Ti) composite (Fig. 1F) showed the distributed particles of Fe_3_O_4_ and MIL-125(Ti) onto the fiber-like particles of κ-Carr, denoting the successful combination between the composite’s components.

#### XRD

**Fig. 2:**
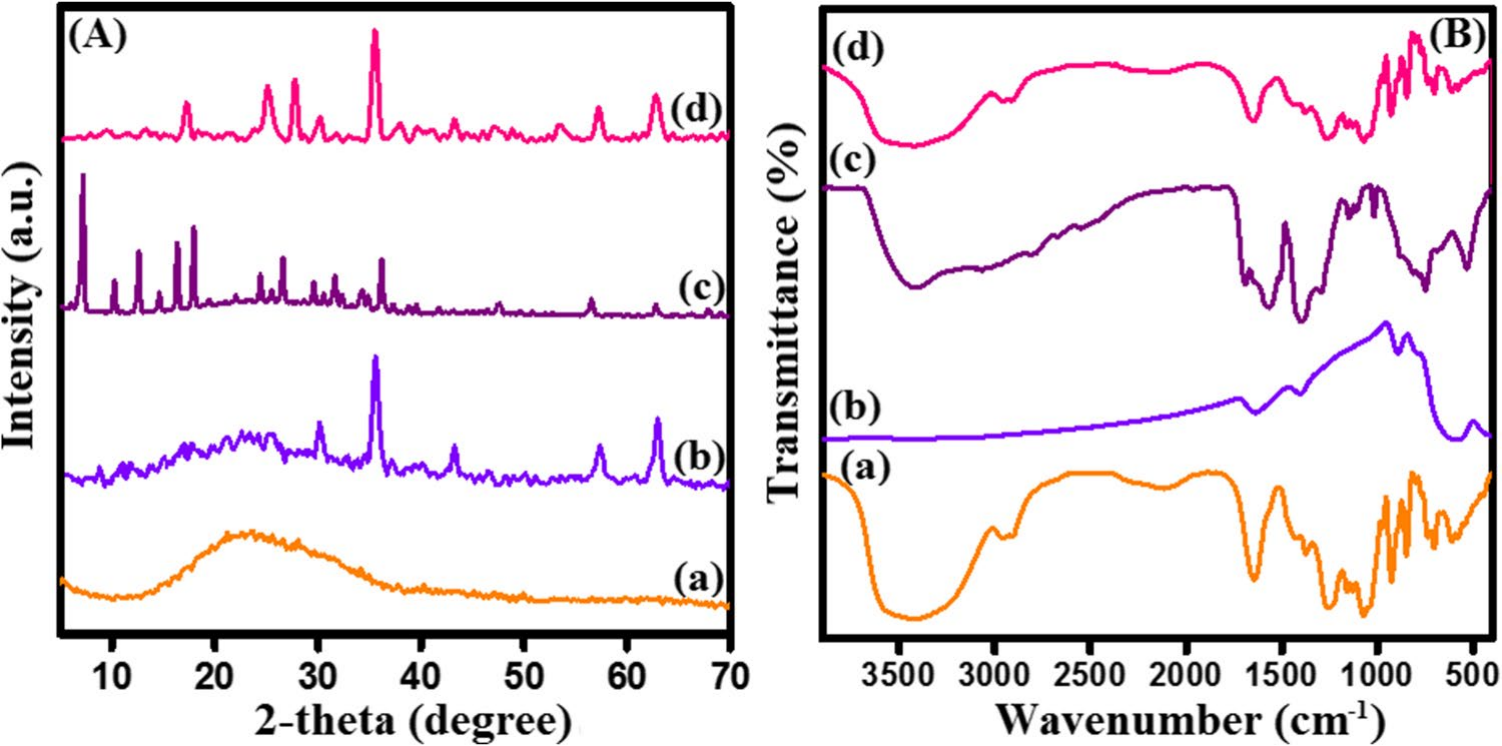
**A** XRD and **B** FTIR of (a) κ-Carr, (b) Fe_3_O_4_, (c) MIL-125, and (d) Fe3O4-κ-Carr/MIL-125(Ti)

The crystallite phases of Fe_3_O_4_, κ-Carr, MIL-125(Ti), and Fe_3_O_4_-κ-Carr/MIL-125(Ti) composite were analyzed by XRD (Fig. 2A). The XRD pattern of κ-Carr signalizes its characteristic broad band around 2*θ* = 20° (Rhim and Wang 2014). Moreover, the XRD pattern of Fe_3_O_4_ elucidates its discriminative peaks at 2*θ* = 30.01, 35.92, 42.48, 57.20, and 62.08° which are corresponded to (220), (311), (400), (511), and (440), respectively (Loh et al. 2008). The XRD pattern reveals the crystalline structure of MIL-125(Ti) since the distinguishing peaks appeared at 2*θ* = 7.11, 10.17, 12.51, 14.53, 16.51, 17.77, 24.57, 26.49, 29.49. 31.79, 34.13, 36.29, 39.49, 47.53, 56.39, and 62.71° (Omer et al. 2021). The XRD pattern of Fe_3_O_4_-κ-Carr/MIL-125(Ti) composite illustrates the characteristic peaks to the pure components but with a diminution in the crystallinity which is most likely due to the amorphous character of κ-Carr.

#### FTIR

Figure 2B depicts the FTIR spectra of κ-Carr, Fe_3_O_4_, MIL-125, and Fe_3_O_4_-κ-Carr/MIL-125(Ti) composite. For κ-Carr, the peaks at 847 and 1260 cm^−1^ are assigned to C-O-S and O = S = O, respectively (Khoshkho et al. 2021). In addition to the peak belonging to C–O–C in 3, 6-anhydrogalactose appeared at 928 cm^−1^ (Arof et al. 2010). The peaks at 1071 and 1128 cm^−1^ are ascribed to S–O and C-O, respectively. While the related peaks of OH and C-H appeared at 3435 and 2909 cm^−1^, respectively (Khoshkho et al. 2021). For pristine Fe_3_O_4_, the characteristic peaks of Fe–O stretching manifested at 557 and 1405 cm^−1^. In addition, the absorption peaks at 892 and 1639 cm^−1^ are ascribed to OH vibrating and OH bending modes, respectively, while the band at 3437 cm^−1^ belongs to OH stretching vibration (Cheng et al. 2021). For MIL-125(Ti), the sharp absorbance peak at 1398 cm^−1^ corresponds to OH of BDC. Furthermore, the characteristic peaks of symmetric C = O of BDC appeared at 1398 cm^−1^ and the asymmetric C = O manifested at 1509 and 1564 cm^−1^ (Jin et al. 2015). In addition, the peaks between 1400 and 1600 cm^−1^ are ascribed to COO of BDC, and the peak at 749 cm^−1^ is attributed to Ti–O (Moreira et al. 2018; Omer et al. 2021). For Fe_3_O_4_-κ-Carr/MIL-125(Ti) composite, the FTIR spectrum reveals the discriminative peaks of κ-Carr, Fe_3_O_4_, and MIL-125(Ti) with slight peaks shifting and lower peaks intensity, suggesting the successful combination between the pure components.

#### XPS

**Fig. 3:**
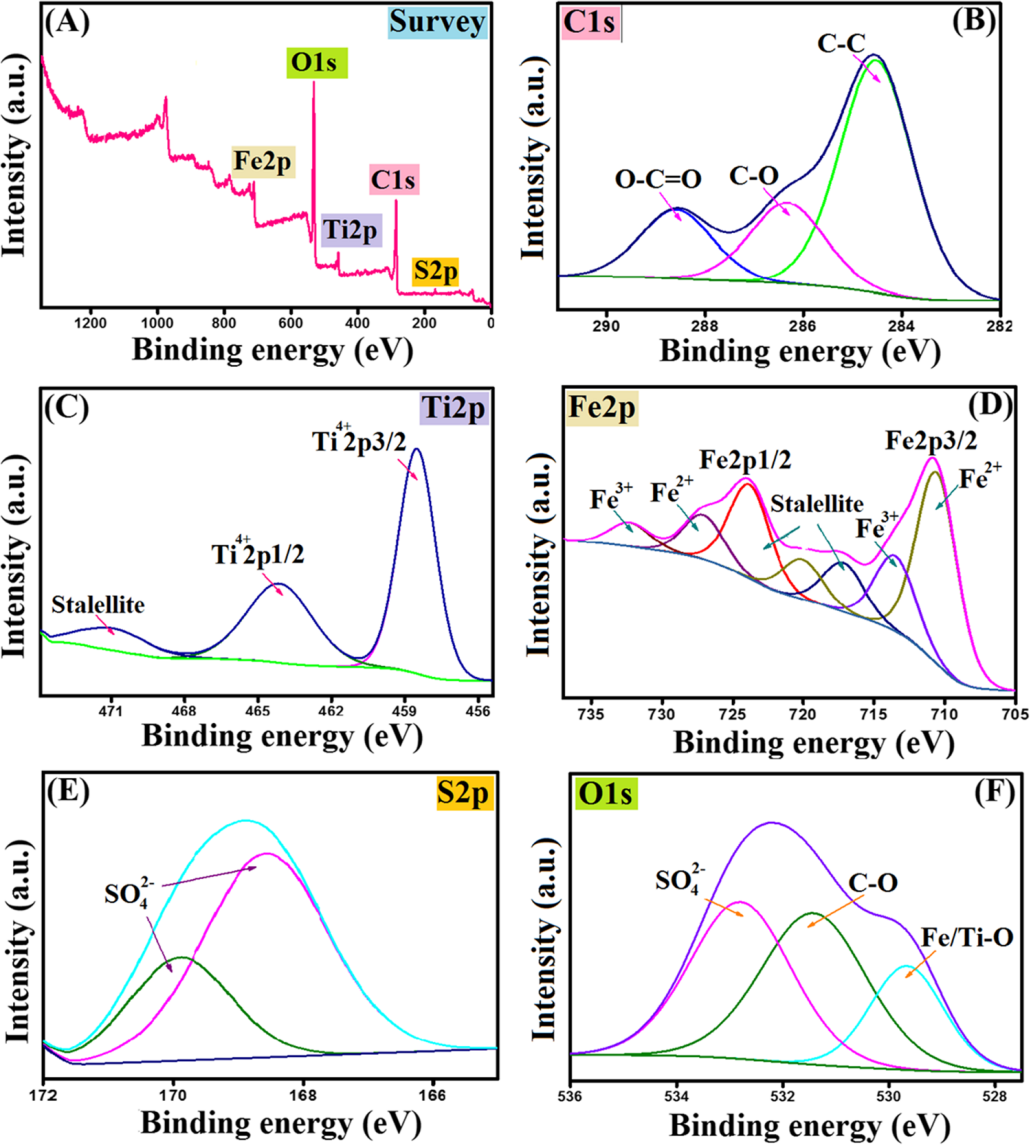
XPS spectra of Fe_3_O_4_-κ-Carr/MIL-125(Ti). **A** Survey, **B** C1s, **C** Ti2p, **D** Fe2p, **E** S2p, and **F** O1s

XPS spectra inferred the incorporation of MIL-125(Ti) and Fe_3_O_4_ into the Carr matrix. The XPS survey (Fig. 3A) implied that Fe_3_O_4_-κ-Carr/MIL-125(Ti) composite consists from C1s, Ti2p, Fe2p, S2p, and O1s. The C1s-spectrum (Fig. 3B) signalized the characteristic peaks of C-O, C–C, and O-C = O at 286.32, 284.52, and 288.59 eV, respectively (Cheng et al. 2022). Moreover, the Ti2p-spectrum (Fig. 3C) illustrated the peaks at 464.12, 470.94, and 458.50 eV which are corresponded to Ti^4+^2p1/2, satellite, and Ti^4+^2p3/2, respectively. The Fe2p-spectrum (Fig. 3D) revealed the presence of Fe^2+^ and Fe^3+^ since the distinguishing peaks to Fe^2+^ appeared at 710.55 and 727.09 eV, while the belonging peaks to Fe^3+^ manifested at 713.50 and 732.28 eV. Furthermore, the related peaks to SO_4_^2−^ group of Carr appeared at 168.83 and 170.10 eV (Fig. 3E). The O1s-spectrum (Fig. 3F) showed the containing oxygen-functionalized groups onto the Fe_3_O_4_-κ-Carr/MIL-125(Ti) backbone since the peaks at 532.80, 531.42, and 529.66 eV are assigned to SO_4_^2−^, Fe–O/Ti–O, and C-O, respectively.

#### VSM

**Fig. 4:**
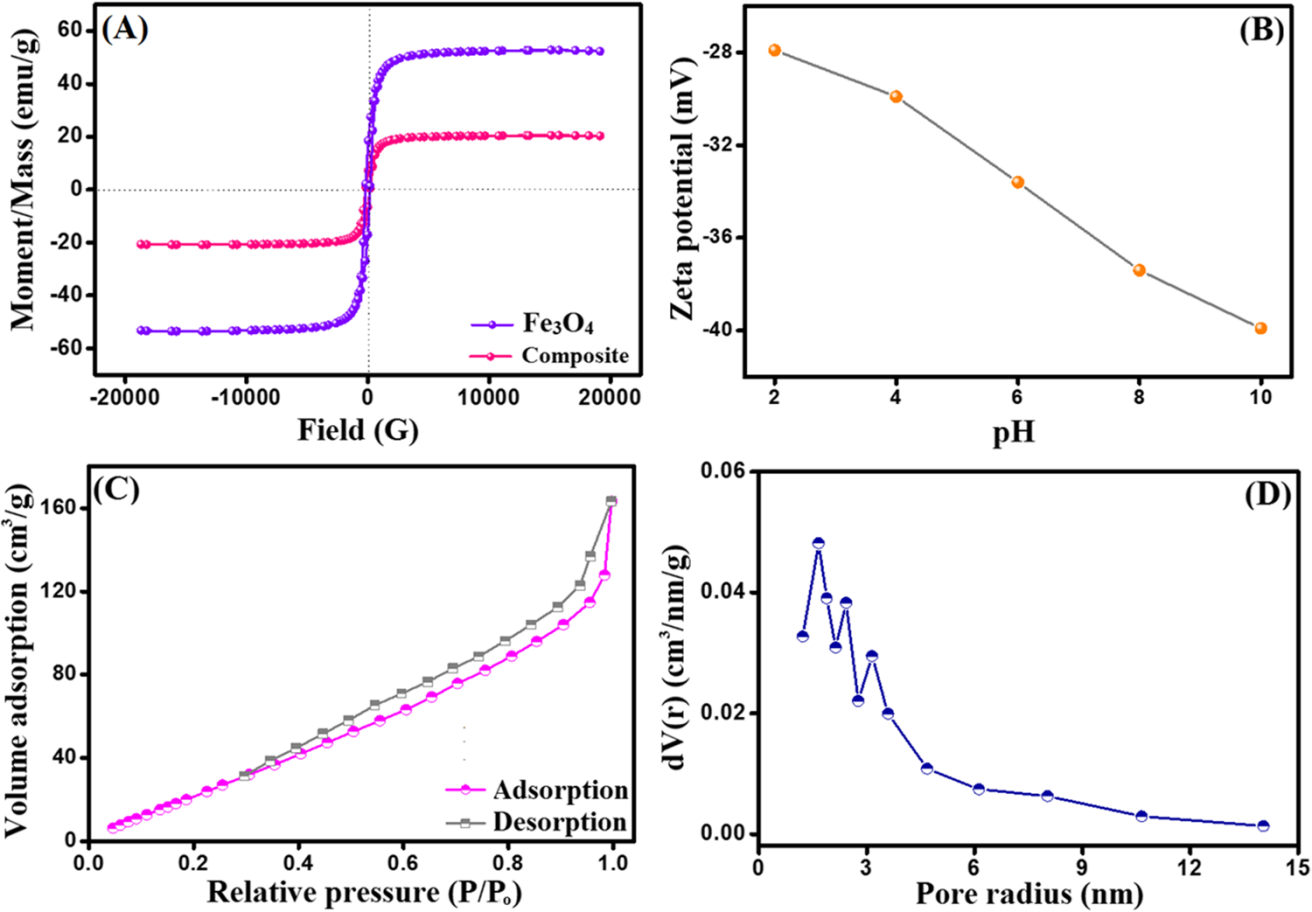
**A** VSM of Fe_3_O_4_ and Fe_3_O_4_-κ-Carr/MIL-125(Ti), **B** ZP of Fe_3_O_4_-κ-Carr/MIL-125(Ti), **C** N_2_-adsorption/desorption isotherm, and **D** the pore size distribution of Fe_3_O_4_-κ-Carr/MIL-125(Ti)

VSM hysteresis loops (Fig. 4A) confirmed the ferromagnetic property of both Fe_3_O_4_ and Fe_3_O_4_-κ-Carr/MIL-125(Ti) composite since the coercivity values were 199.86 and 94.56 G, respectively. Moreover, an expected decline in the saturation magnetization of Fe_3_O_4_ (Ms = 52.34 emu/g) occurred which is most likely due to its blinding with the non-magnetic Carr and MIL-125(Ti). Notably, Fe_3_O_4_-κ-Carr/MIL-125(Ti) composite possesses a propitious magnetic property (Ms = 20.34 emu/g), endowing it the easy separation advantage by an external magnet instead of the conventional techniques that consume a long time.

#### ZP measurements

Figure 4B depicts the ZP measurements of Fe_3_O_4_-κ-Carr/MIL-125(Ti) composite at a pH ranging from 3 to 11. It is apparent that Fe_3_O_4_-κ-Carr/MIL-125(Ti) composite displayed a negatively charged surface, while the ZP value was amplified from − 27.9 to − 39.9 mV with a raising of the pH from 2 to 10, respectively. This finding suggests the suitability of Fe_3_O_4_-κ-Carr/MIL-125(Ti) to adsorb zwitter ionic, neutral, and cationic pollutants.

#### BET

The N_2_-adsorption/desorption isotherm (Fig. 4C) elucidated that Fe_3_O_4_-κ-Carr/MIL-125(Ti) composite showed a type II with H_4_-type hysteresis loop according to the IUPAC classification, suggesting the mesoporous structure of the composite. Moreover, the specific surface area of Fe_3_O_4_-κ-Carr/MIL-125(Ti) composite was 163.27 m^2^/g, and the average pore diameter was 2.861 nm (Fig. 4D).

### Optimization of the o-NP adsorption process

#### Comparison test

**Fig. 5:**
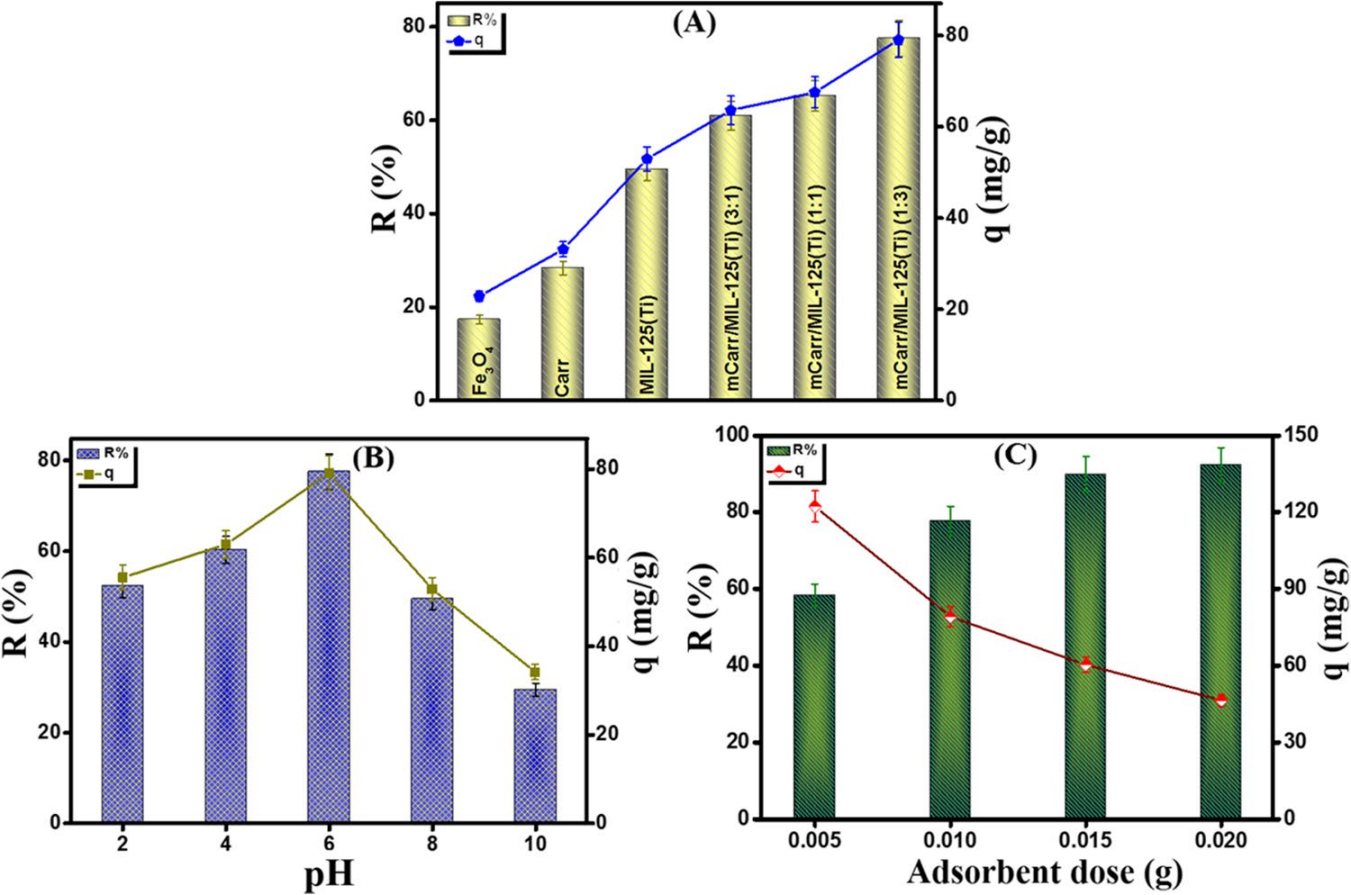
**A** Comparison study [pH = 6, *C*_o_ = 50 mg/L, *m* = 0.01 g, and *T* = 25 °C], **B** the impact of pH medium [pH = 2–10, *C*_o_ = 50 mg/L, *m* = 0.01 g, and *T* = 25 °C], and **C** the impact of the Fe_3_O_4_-κ-Carr/MIL-125(Ti) dose [*m* = 0.005–0.02 g, *C*_o_ = 50 mg/L, pH = 6, and *T* = 25 °C] on the o-NP adsorption

 For assessing the amelioration of the adsorption performance of Carr toward o-NP after blinding with MIL-125(Ti), a comparison test was executed between the pure materials and the three fabricated composites (Fig. 5A). It was found that the removal % of Fe_3_O_4_, κ-Carr, and MIL-125(Ti) were 17.35, 28.35, and 49.55% and the adsorption capacity were 22.86, 33.12, and 52.91 mg/g, respectively. Furthermore, the removal % and the adsorption capacity of Fe_3_O_4_-κ-Carr/MIL-125(Ti) composites with κ-Carr: MIL-125(Ti) ratios 3:1, 1:1, and 1:3 were 60.99, 65.19, and 77.55% and 63.60, 67.51, and 79.05 mg/g, respectively (Table S1). In light of these results, the modification of κ-Carr with efficient material like MIL-125(Ti) is an effective approach as it increased the removal % of o-NP by more than 2.5-fold. In addition to the dual function of Fe_3_O_4_ that provides perfect separation and enhances the adsorption aptitude of o-NP, the Fe_3_O_4_-κ-Carr/MIL-125(Ti) composite with a ratio of 1:3 between κ-Carr and MIL-125(Ti) was chosen for the rest of the batch experiments.

#### Effect of the solution pH

In general, pH is the dominant parameter in the uptake processes, so the o-NP uptake onto Fe_3_O_4_-κ-Carr/MIL-125(Ti) composite was investigated at a wide scale of pH media (Fig. 5B). The experimental results indicated the superiority of the o-NP adsorption onto Fe_3_O_4_-κ-Carr/MIL-125(Ti) at pH 6. This finding could be explained by the pKa of o-NP = 7.23, meaning that o-NP exists in the molecular form in acidic conditions (Ma et al. 2019; Marques et al. 2020). Thereby, the electrostatic interaction is not the controlling mechanism on the adsorption of o-NP onto Fe_3_O_4_-κ-Carr/MIL-125(Ti), and there are other chemical and physical interactions such as *π*-*π* interaction, H-bonding, and electron donor–acceptor interaction could take place between o-NP and Fe_3_O_4_-κ-Carr/MIL-125(Ti) in the acidic medium (Chen et al. 2017). Conversely, it was observed a dramatic diminution in the o-NP adsorption aptitude when pH > 6 since the removal % and the adsorption capacity of o-NP dwindled from 77.55% and 79.05 mg/g to 26.02% and 34.18 mg/g. This finding may be anticipated by the strong repulsion forces between the anionic o-NP and the negatively charged Fe_3_O_4_-κ-Carr/MIL-125(Ti) composite (Liu et al. 2020).

#### The effect of the adsorbent dose

Figure 5C represents the impact of the dosage of Fe_3_O_4_-κ-Carr/MIL-125(Ti) composite onto the adsorption efficiency o-NP. It is apparent that the augmentation in the composite dose from 0.005 to 0.02 g causes an increase in the removal % of o-NP from 58.28 to 92.29%, respectively, which is most likely due to the increase in the number of active sites. On the contrary, a decline in the adsorption capacity of o-NP from 122.12 to 46.40 mg/g was observed with the raising in the Fe_3_O_4_-κ-Carr/MIL-125(Ti) dose which may be attributed to the aggregation of the extra amount of the composite, resulting in a diminution in the surface area.

#### The effect of ionic strength

**Fig. 6:**
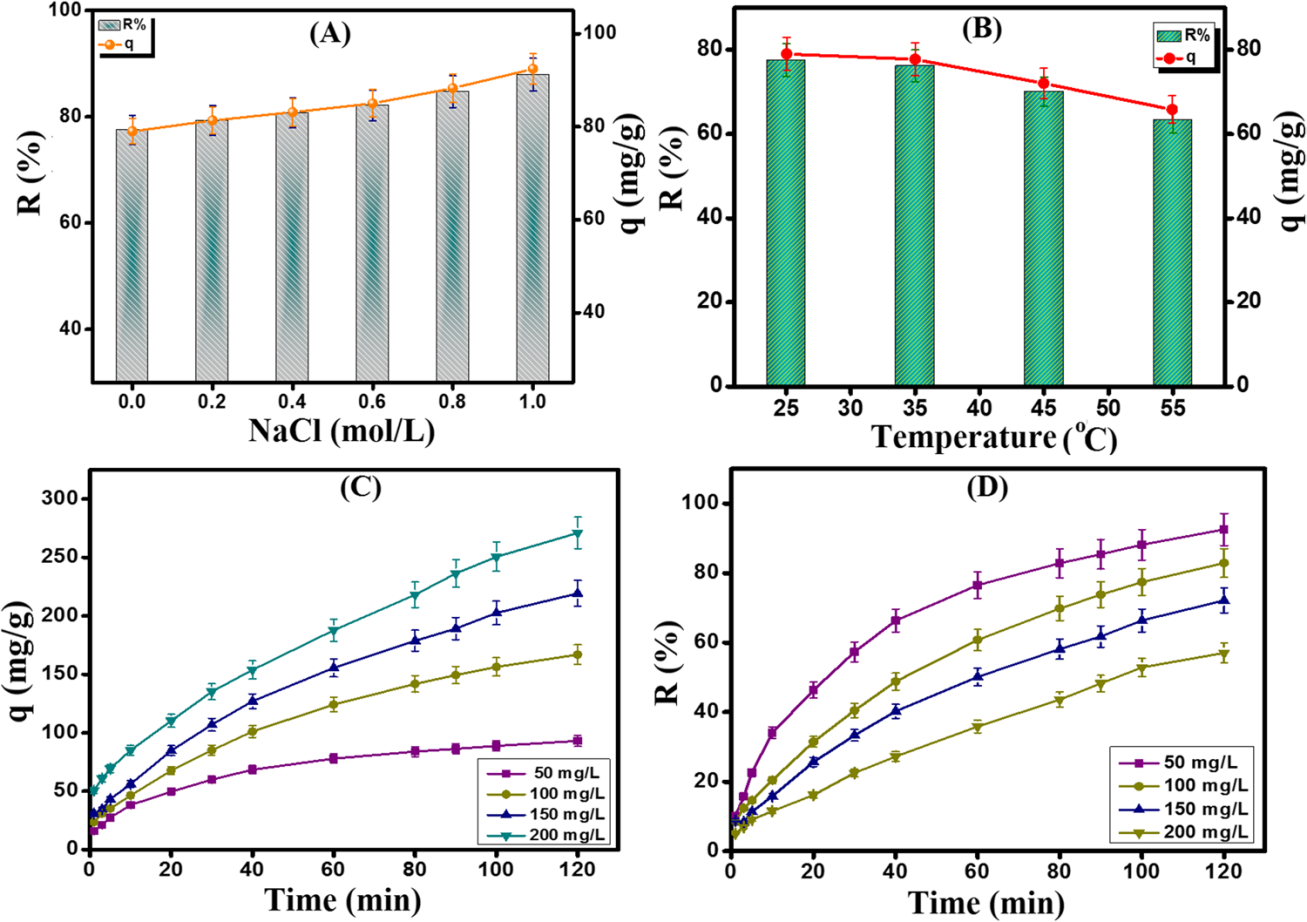
**A** Effect of ionic strength [NaCl concentration = 0.2–1.0 mol/L, pH = 6, *C*_o_ = 50 mg/L, *m* = 0.01 g, and *T* = 25 °C], **B** effect of process temperature [*T* = 25–55 °C, pH = 6, *C*_o_ = 50 mg/L, and *m* = 0.01 g], and **C**, **D** effect of initial concentration of o-NP [*C*_o_ = 50–200 mg/L, *T* = 25 °C, pH = 6, and *m* = 0.01 g] on the adsorption of o-NP onto Fe_3_O_4_-κ-Carr/MIL-125(Ti) composite

Figure 6A reveals the impact of the ionic strength on the o-NP adsorption onto Fe_3_O_4_-κ-Carr/MIL-125(Ti) composite. It was recorded an enhancement in the adsorption aptitude of o-NP with the increase in the NaCl concentration from 0.2 to 1.0 mol/L since the adsorption capacity and removal % incremented from 79.31% and 81.33 mg/g to 87.93% and 92.45 mg/g, respectively. This behavior is most likely due to the salting out effect as the presence of NaCl declines the o-NP solubility, agreeing with Mengzhi Yang et al. 2018 (Yang and Wang 2018).

#### The effect of the temperature

It was deduced the exothermal nature of the o-NP adsorption onto Fe_3_O_4_-κ-Carr/MIL-125(Ti) since the adsorption capacity and removal % of o-NP dwindled from 79.05 mg/g and 77.55% to 65.82 mg/g and 63.38% with raising the process temperature from 25 to 55 °C, respectively (Fig. 6B). This behavior may be assigned to the increase in the system temperature causes an increment in the Brownian motion of the o-NP molecules inside the bulk solution. Hence, the adsorption aptitude o-NP onto the Fe_3_O_4_-κ-Carr/MIL-125(Ti) surface directly diminished.

#### The effect of the initial concentration

Figure 6C depicts the influence of the increment of the initial concentration of the bulk solution on the adsorption efficacy of o-NP. It was found that the increase in the o-NP concentration from 50 to 200 mg/g caused an increase in the adsorption capacity from 93.02 to 271.11 mg/g. This finding may be explained by the concentration increase in the bulk solution, generating strong driving forces of o-NP toward the Fe_3_O_4_-κ-Carr/MIL-125(Ti) surface. Thence, such potent forces could overcome the mass transfer resistance to the migration of o-NP from the bulk solution to Fe_3_O_4_-κ-Carr/MIL-125(Ti) surface (Gomaa et al. 2022a). On the contrary, this increase in the concentration of o-NP solution resulted in a diminution in the removal % from 92.54 to 57.05%, which is most likely due to the inadequate binding sites into the Fe_3_O_4_-κ-Carr/MIL-125(Ti) surface (Fig. 6D) (Eltaweil et al. 2022c). Overall, Fe_3_O_4_-κ-Carr/MIL-125(Ti) composite not only exhibited efficient adsorption performance toward the detrimental o-NP but also fast adsorption since the equilibrium time was 60 min.

### Kinetic study

**Fig. 7:**
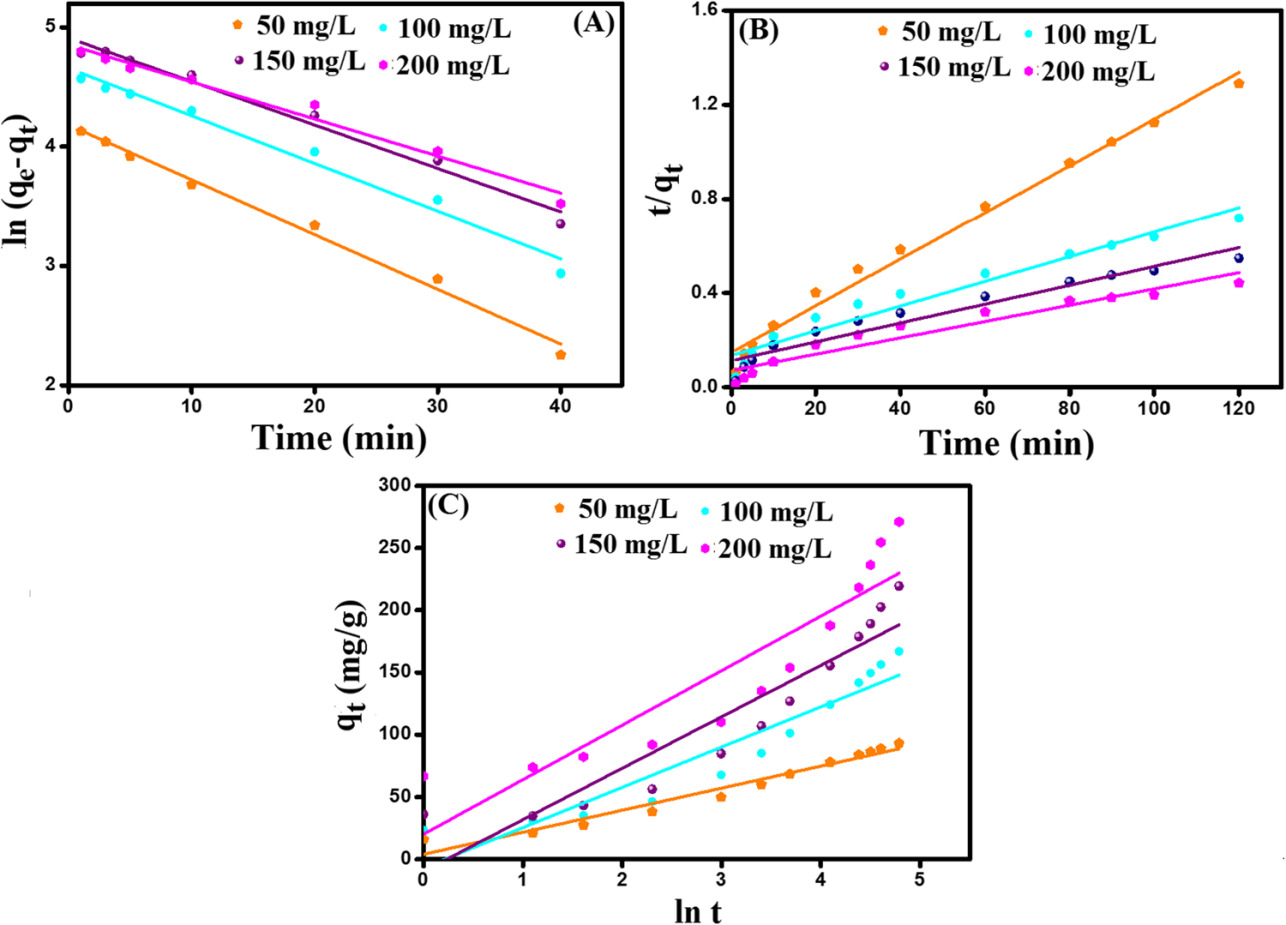
Kinetic study **A** PFO, **B** PSO, and **C** Elovich models

The experimental results pointed out that the adsorption of o-NP onto Fe_3_O_4_-κ-Carr/MIL-125(Ti) composite may occur via diverse mechanisms, depending on the heterogeneity of the binding sites onto the composite surface and physicochemical conditions. Therefore, various kinetic models: pseudo-first-order (PFO), pseudo-second-order (PSO), and Elovich, were utilized to analyze the resultant experimental data (Fig. 7A-C). The linear expressions of the applied models are listed in Table S2.

It was deduced from the computed *R*^2^ values (Table 1) that PSO is more suitable than PFO to represent the o-NP adsorption onto Fe_3_O_4_-κ-Carr/MIL-125(Ti) composite since *R*^2^-PSO > *R*^2^-PFO (Eltaweil et al. 2022b). Furthermore, the obtained *q*_cal_ from PSO are closer to *q*_exp_ than those calculated from PFO. Besides, the favorability of the o-NP adsorption process was proved by Elovich model since the rate of o-NP adsorption onto Fe_3_O_4_-κ-Carr/MIL-125(Ti) was greater than the desorption rate.

**Table 1:** The derived parameters from PFO, PSO, and Elovich kinetic models of the adsorption of o-NP onto Fe_3_O_4_-κ-Carr/MIL-125(Ti) composite

Kinetic models and parameters	Concentration (mg/L)			
	50	100	150	200
*q*_*e*, exp_ (mg/g)	93.02	166.99	219.37	271.11
PFO				
* q*_*e*,cal_ (mg/g)	65.37	104.58	134.29	172.74
* k*_1_ (min^−1^)	0.046	0.039	0.036	0.031
* R*^2^	0.989	0.977	0.979	0.975
PSO				
* q*_*e*,cal_ (mg/g)	100.90	190.84	250.00	289.02
* k*_2_ (g.mg^−1^.min^−1^)	0.0007	0.0004	0.0002	0.0001
* R*^2^	0.997	0.987	0.983	0.985
Elovich				
* α* (mg/g min)	22.29	26.04	33.11	72.02
* β* (g/mg)	0.056	0.031	0.024	0.022
* R*^2^	0.958	0.888	0.863	0.820

### Isotherm study

**Fig. 8:**
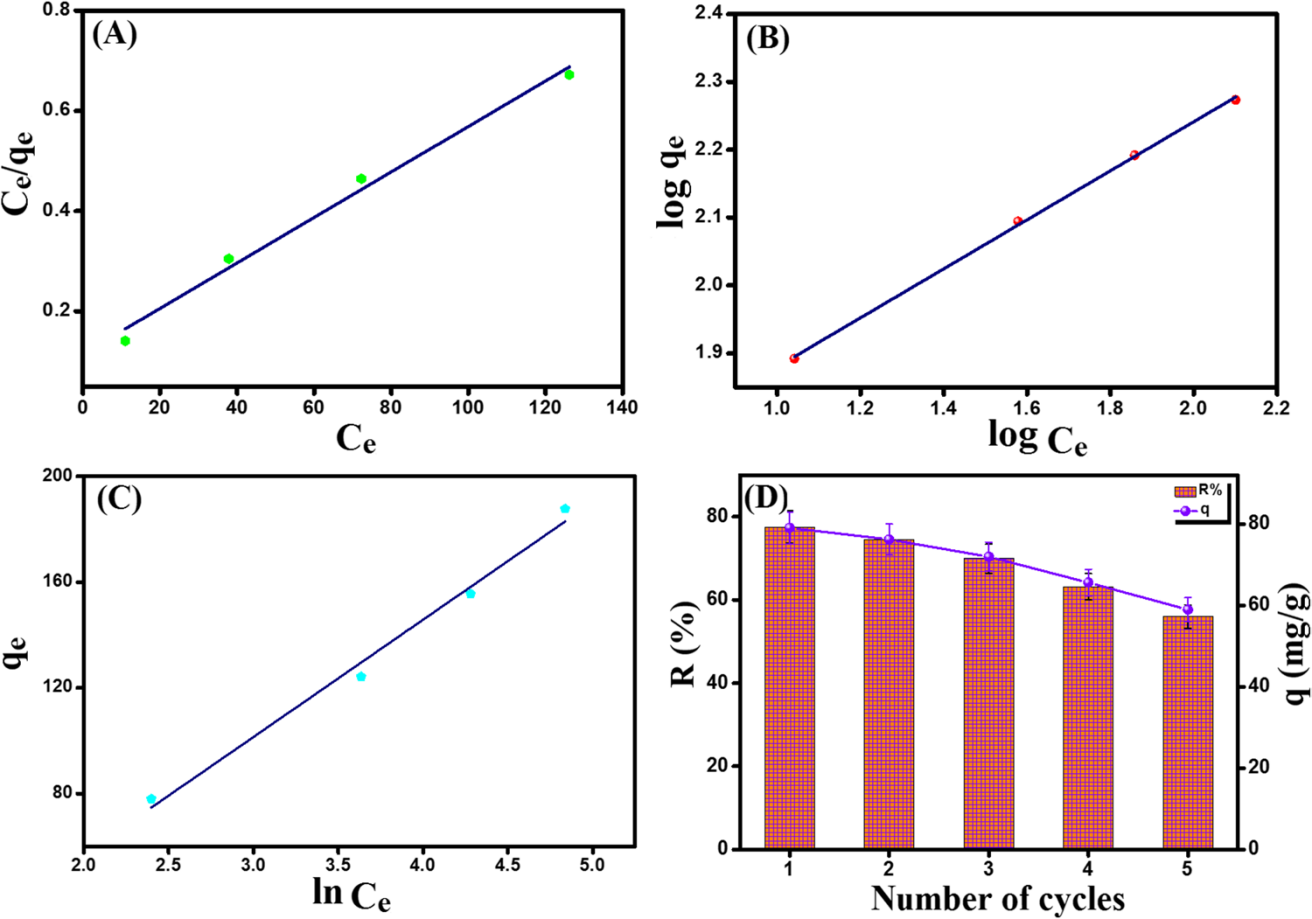
Isotherm study. **A** Langmuir, **B** Freundlich, and **C** Temkin models, and **D** recyclability test [NaOH concentration = 1 M, pH = 6, *C*_o_ = 50 mg/L, *m* = 0.01 g, and *T* = 25 °C]

The type of interactions between o-NP and Fe_3_O_4_-κ-Carr/MIL-125(Ti) composite at equilibrium was scrutinized by bountiful isotherm models, including Langmuir, Freundlich, and Temkin (Fig. 8A–C). The linear expressions of these models were summarized in Table S3.

The obtained isotherm parameters (Table 2) point out that Freundlich model (*R*^2^ = 0.999) is more fitted than Langmuir (*R*^2^ = 0.984) and Temkin (*R*^2^ = 0.983) to model the equilibrium data of the o-NP adsorption onto Fe_3_O_4_-κ-Carr/MIL-125(Ti). Additionally, the *b* value from Temkin model evinced the same result since *b* < 80 kJ/mol. Moreover, it was found that *n* > 2 reflects the favorability of the o-NP adsorption process onto Fe_3_O_4_-κ-Carr/MIL-125(Ti) composite. Furthermore, the calculated *q*_max_ of o-NP onto Fe_3_O_4_-κ-Carr/MIL-125(Ti) composite under Langmuir was 320.26 mg/g at pH 6 and 25 °C.

**Table 2:** The parameters derived from Langmuir, Freundlich, and Temkin isotherm models for the adsorption of o-NP onto Fe_3_O_4_-κ-Carr/MIL-125(Ti) composite

Isotherm model	Parameter	Value
Langmuir	*q*_*m*_ (mg/g)	320.26
	*b* (L/mg)	0.027
	*R*^2^	0.984
Freundlich	*n*	2.77
	*k*_F_ (L/mg)	14.18
	*R*^2^	0.999
Temkin	*A* (L/g)	0.489
	*B* (J/mol)	44.36
	*b* (KJ/mol)	0.051
	*R*^2^	0.983

### Reusability study

It is apparent from the recyclability test (Fig. 8D) that Fe_3_O_4_-κ-Carr/MIL-125(Ti) composite has remarkable recyclability where its adsorption performance toward o-NP was still high after the 5th cycle (*q* = 58.94 mg/g and *R*% = 56.01%). This finding confirmed the significance of these magnetic adsorbents that provide a fast, easy, and impeccable separation after the adsorption processes using an external magnet.

### The proposed adsorption mechanism

**Fig. 9:**
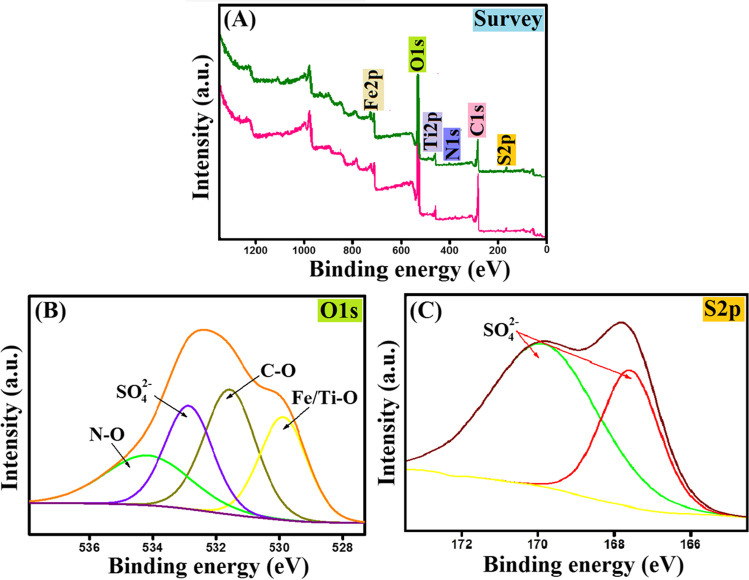
XPS spectra of Fe_3_O_4_-κ-Carr/MIL-125(Ti); **A** survey before and after the adsorption of o-NP, **B** O1s, and **C** S2p after the adsorption process

Understanding the adsorption mechanism is a quintessential point in any adsorption process, so the controlled mechanism on the adsorption of o-NP onto Fe_3_O_4_-κ-Carr/MIL-125(Ti) composite was studied based on XPS analysis, ZP measurements, and the experimental results. XPS survey of Fe_3_O_4_-κ-Carr/MIL-125(Ti) after the adsorption of o-NP (Fig. 9A) showed the characteristic peak of N1s, evincing the adsorption of o-NP. The presence of o-NP in the molecular form at pH < 7.23 suggested that the electrostatic interaction is not the dominant mechanism, and other physical and chemical interactions that played positive and negative effects on the o-NP adsorption process, including (i) H-bonding between the N and O-containing groups on the o-NP and the H-atoms of Fe_3_O_4_-κ-Carr/MIL-125(Ti) as well as the plentiful O-containing groups onto the Fe_3_O_4_-κ-Carr/MIL-125(Ti) surface and the H-atoms of o-NP; (ii) *π*-*π* interaction between the aromatic ring of BDC in the composite and the benzene ring in o-NP; (iii) electron donor–acceptor interaction between the e-donor groups in Fe_3_O_4_-κ-Carr/MIL-125(Ti) composite (OH, SO_4_^2−^ and benzene ring) and the e-withdrawing group in o-NP (NO_2_) as well as the e-withdrawing group in the composite (COOH) and the e-donor groups in o-NP (OH and benzene ring). The occurrence of these physicochemical interactions between o-NP and Fe_3_O_4_-κ-Carr/MIL-125(Ti) was confirmed by the peak shift of the XPS spectra of O1s and S2p after the o-NP adsorption (Fig. 9B, C). (iv) The electrostatic repulsion forces between the anionic o-NP and the negatively charged composite as clarified from ZP measurements in an alkaline medium, played a secondary negative role on the adsorption aptitude of o-NP.

## Conclusion

This study reported the construction, characterization, and adsorbability evaluation of a new Fe_3_O_4_-κ-Carr/MIL-125(Ti) composite for removing the organic o-NP pollutant. The characterization stage inferred the successful formulation of the composite adsorbent. Surprisingly, increasing MIL-125(Ti) ratio three times than κ-Carr significantly boosted the removal (%) of o-NP from 60.99 to 77.55%, and the maximal adsorption capacity of o-NP attained 320.26 mg/g at pH 6 and 25 ^o^C. Moreover, data obtained from kinetics and isotherm studies were fitted to Freundlich model and followed the pseudo-second-order model. The reusability test attested the potential capability of Fe_3_O_4_-κ-Carr/MIL-125(Ti) composite to adsorb o-NP after five repeated cycles with removal (%) exceeded 60%. Overall, the higher adsorption performance, facile separation, and recyclability features nominate the potential use of the formulated composite as an efficient adsorbent candidate for advanced water treatment.

## Supplementary Information

Below is the link to the electronic supplementary material.

Supplementary file1 (DOCX 17 KB)

## Data Availability

The datasets used and analyzed during the current study are available from the corresponding author on reasonable request.
