# Supplementary material for: Adsorption of nitrophenol onto a novel Fe3O4-κ-carrageenan/MIL-125(Ti) composite: process optimization, isotherms, kinetics, and mechanism
Source: Environ Sci Pollut Res Int. 2023 Feb 11;30(17):49301–13. doi: 10.1007/s11356-023-25678-2 (PMC10104928; doi:10.1007/s11356-023-25678-2)
Supplement: Supplementary file 1 — Supplementary file1 (DOCX 17 KB) [file 11356_2023_25678_MOESM1_ESM.docx]

**Adsorption of nitrophenol onto a novel Fe_3_O_4_-κ-carrageenan/MIL-125(Ti) composite; process optimization, Isotherms, Kinetics and Mechanism**

Eman M. Abd El-Monaem^a,*^, Abdelazeem S. Eltaweil^a,^, Gehan M. El-Subruiti^a^, Mohamed S. Mohy-Eldin^b^ and Ahmed M. Omer^b^
^a^Chemistry Department, Faculty of Science, Alexandria University, Alexandria, Egypt.

^b^Polymer Materials Research Department, Advanced Technology and New Materials Research Institute (ATNMRI), City of Scientific Research and Technological Applications (SRTA-City), New Borg El-Arab City, P. O. Box: 21934, Alexandria, Egypt

*Correspondence: Eman M. Abd El-Monaem ([emanabdelmonaem5925@yahoo.com](mailto:emanabdelmonaem5925@yahoo.com))

Tel.: +20-3-4593414

**Table S1:** The results of the comparison test between Fe_3_O_4_-κ-Carr/MIL-125(Ti) composites and the pristine components toward the o-NP adsorption.

| **Adsorbent** | **R (%)** | **q (mg/g)** |
| --- | --- | --- |
| Fe_3_O_4_ | 17.35 | 22.86 |
| κ-Carr | 28.35 | 33.12 |
| MIL-125(Ti) | 49.55 | 52.91 |
| Fe_3_O_4_-κ-Carr/MIL-125(Ti)  (κ-Carr:MIL-125(Ti)= 3:1) | 60.99 | 63.60 |
| Fe_3_O_4_-κ-Carr/MIL-125(Ti)  (κ-Carr:MIL-125(Ti)= 1:1) | 65.19 | 67.51 |
| Fe_3_O_4_-κ-Carr/MIL-125(Ti)  (κ-Carr:MIL-125(Ti)= 1:3) | 77.55 | 79.05 |

**Table S2:** Non-linear equations of the applied adsorption kinetic models.

| **Kinetic Model** | **Equation** |
| --- | --- |
| **PFO** | $q_{t}=q_{e}\left( 1-e^{-k_{1}t} \right)$(1) |
| **PSO** | $q_{t}=\frac{tk_{2}q_{e}^{2}}{1+t k_{2}q_{e}}$ (2) |
| **Elovich** | $q_{t}=\frac{1}{\beta}\ln(\alpha\beta t+1)$ (3) |

Where, q_t_ and q_e_ are amount of o-NP uptakes at time t and equilibrium, respectively. k_1_ and k_2_ are the rate constants of PFO and PSO, respectively. Furthermore, α and β are Elovich coefficients that represent the initial adsorption rate and the desorption coefficient, respectively, also related to the extent of surface coverage and activation energy for chemisorption.

**Table S3:** Non-linear equations of the applied adsorption isotherm models.

| **Model** | **Equation** |
| --- | --- |
| **Langmuir** | $q_{e}=\frac{q_{max}K_{L}C_{e}}{1+ K_{L} C_{e}}$ (3) |
| **Freundlich** | $q_{e}=k_{F}C_{e}^{1/n}$ (4) |
| **Temkin** | $q_{e}=\frac{R T}{b_{T}}\ln k_{T}C_{e}$ (5) |

Where, q_m_ and K_L_ are the maximum monolayer adsorption capacity and Langmuir constant, respectively. n and K_F_ are Freundlich constants. A is the equilibrium binding constant and $B=\frac{\mathrm{RT}}{b}$ , b is Temkin constant related to heat of adsorption.
